# Three-stage hybrid spiking neural networks fine-tuning for speech enhancement

**DOI:** 10.3389/fnins.2025.1567347

**Published:** 2025-04-30

**Authors:** Nidal Abuhajar, Zhewei Wang, Marc Baltes, Ye Yue, Li Xu, Avinash Karanth, Charles D. Smith, Jundong Liu

**Affiliations:** ^1^School of Electrical Engineering and Computer Science, Ohio University, Athens, OH, United States; ^2^Department of Hearing, Speech, and Language Sciences, Ohio University, Athens, OH, United States; ^3^Department of Neurology, University of Kentucky, Lexington, KY, United States

**Keywords:** spiking neural network (SNN), Wave-U-Net, speech enhancement, Conv-TasNet, ANN-SNN conversion

## Abstract

**Introduction:**

In the past decade, artificial neural networks (ANNs) have revolutionized many AI-related fields, including Speech Enhancement (SE). However, achieving high performance with ANNs often requires substantial power and memory resources. Recently, spiking neural networks (SNNs) have emerged as a promising low-power alternative to ANNs, leveraging their inherent sparsity to enable efficient computation while maintaining performance.

**Method:**

While SNNs offer improved energy efficiency, they are generally more challenging to train compared to ANNs. In this study, we propose a three-stage hybrid ANN-to-SNN fine-tuning scheme and apply it to Wave-U-Net and ConvTasNet, two major network solutions for speech enhancement. Our framework first trains the ANN models, followed by converting them into their corresponding spiking versions. The converted SNNs are subsequently fine-tuned with a hybrid training scheme, where the forward pass uses spiking signals and the backward pass uses ANN signals to enable backpropagation. In order to maintain the performance of the original ANN models, various modifications to the original network architectures have been made. Our SNN models operate entirely in the temporal domain, eliminating the need to convert wave signals into the spectral domain for input and back to the waveform for output. Moreover, our models uniquely utilize spiking neurons, setting them apart from many models that incorporate regular ANN neurons in their architectures.

**Results and discussion:**

Experiments on noisy VCTK and TIMIT datasets demonstrate the effectiveness of the hybrid training, where the fine-tuned SNNs show significant improvement and robustness over the baseline models.

## 1 Introduction

Speech enhancement (SE) is a fundamental problem in speech signal processing, aimed at separating noise from speech to produce a cleaner and more intelligible audio signal. SE plays a critical role in various applications, including hearing aids, speech recognition, and telecommunications. In recent years, deep artificial neural networks (ANNs) have emerged as the dominant solution for SE tasks, producing state-of-the-art results.

However, the advancements of ANNs come at the cost of increased power consumption, primarily due to the complexity of models with deeper learning layers, which limits their applications in many power-constrained scenarios. Spiking neural networks (SNNs) offer a promising solution to this issue. The spiking neurons in SNNs are designed to emulate the temporal and spiking behavior of biological neurons (Bouvier et al., 2019). Unlike traditional neurons, spiking neurons consume energy only when emitting spikes, resulting in a significant reduction in the overall power consumption of the network.

Compared to training ANNs with digital values, training SNNs with binary values is significantly more challenging. Various approaches have been proposed recently to tackle this issue. One common approach is to train SNNs from scratch using surrogate gradients (Lee et al., 2016), which approximate gradients by replacing spike activation function with a smooth curve to overcome the non-differentiability issue of spikes. While widely used, this approach can be inefficient due to vanishing gradient problems and error accumulation that results from gradient approximation, which often resulting in slow convergence, particularly in networks with deep complex architectures (Shrestha and Orchard, 2018; Wu et al., 2018; Rathi et al., 2020; Li et al., 2021). Moreover, training SNNs from scratch on large datasets, such as ImageNet or LibriSpeech, remains a substantial challenge.

An alternative approach to obtaining a trained SNN is through ANN-SNN conversion, where an ANN is first trained and then transformed into an equivalent SNN. Early works such as Diehl et al. (2015) highlighted the issue of performance degradation in converted SNNs due to improper neural activation and introduced weight normalization to adjust firing rates by scaling ANN weights accordingly. To further improve conversion accuracy, Rueckauer et al. (2017) proposed the Max-Norm algorithm, which scales weights based on the maximum activation within each layer, alongside a reset-by-subtraction mechanism to mitigate performance loss. In addition, Sengupta et al. (2019) developed SpikeNorm, which improves SNN performance through threshold rescaling, ensuring better alignment between ANN activations and SNN firing rates. More recently, Ho and Chang (2021) introduced a method that incorporates clipping layers during ANN training to reduce quantization errors before conversion, leading to improved accuracy in converted SNNs. A major limitation of the aforementioned ANN-SNN conversion methods is the need for a large number of time steps to achieve accuracy comparable to the original ANN. This constraint significantly impacts efficiency, making these approaches less suitable for dense, continuous data such as speech, where low-latency processing is critical.

More recent conversion methods aimed to improve SNN accuracy with low-latency. Bu et al. (2022) proposed training an ANN with quantization clip-floor-shift activation to replace ReLU that aims to reduce the clipping error during SNN conversion. Similarly, Wang et al. (2023) proposed a two-stage conversion method involving training an ANN with a clipping activation in the first stage to reduce the clipping and quantization errors, and performing a layer-wise calibration of weights and membrane potentials in the second stage to reduce residual error. While these methods are effective for sparse prediction tasks such as classification—where some degree of information loss from clipping can be tolerated—they fall short in dense prediction tasks like segmentation or speech denoising. These tasks require predictions at every spatial or temporal location, making them far more sensitive to information loss, particularly in preserving fine-grained temporal details critical for accurate speech prediction.

Combining the direct training and ANN-SNN conversion approaches, Baltes et al. (2023) proposed a hybrid SNN fine-tuning pipeline to achieve comparable performance in SNNs. Similar to the ANN-SNN conversion method, this approach begins by transferring the weights from a fully trained ANN to an equivalent SNN architecture. The converted SNN is then fine-tuned through a hybrid forward-backward training process. In this fine-tuning phase, the forward pass consists of standard SNN inference, while the backward pass uses conventional backpropagation to update the SNN weights. This fine-tuning process helps recover information lost during the conversion, allowing the SNN to regain performance that may have degraded due to quantization errors, or clipping like the aforementioned ANN-SNN conversion techniques.

The focus of this study is to develop SNN solutions for speech enhancement. Numerous ANN models for SE have been proposed over the years. Wave-U-Net (Stoller et al., 2018) and Conv-Tasnet (Luo and Mesgarani, 2019) are among the most popular solutions, demonstrating competitive performance on various datasets and SE tasks. Wave-U-Net is a Fully Convolutional Network (FCN) that employs an encoder-decoder architecture. Conv-TasNet features a more advanced encoder-separator-decoder architecture compared to Wave-U-Net, where the separator is designed to estimate a noise mask, allowing the model to capture long-term long-term dependencies while maintaining efficiency.

Very limited research has been conducted to address the SE problem using spiking networks, with the exception of Riahi and Plourde (2023), Sun and Bohte (2024), and Hao et al. (2024). However, these approaches either operate in the frequency domain or rely on hybrid architectures that combine ANN and SNN neurons, which leads to increased latency and energy consumption.

In this work, we build upon the approach proposed by Baltes et al. (2023) and propose a three-stage SNN training scheme for speech enhancement. ANN models are first trained before being converted into their corresponding spiking versions. The converted SNNs are then fine-tuned using a hybrid training scheme, where the forward pass employs spiking signals, while the backward pass utilizes ANN signals to enable backpropagation. We apply thie approach to two major SE netwroks solutions: Wave-U-Net and Conv-TasNet. The contributions made in this work can be summarized as follows:

1. We propose a novel application of a three-stage SNN training scheme, originally developed for image classification tasks, to the regression-based speech enhancement task. We apply our pipeline to two major SE networks, Wave-U-Net, and Conv-TasNet.
2. To the best of our knowledge, this is the first SNN implementation of Conv-TasNet, which is considered one of the state-of-the-art convolutional architectures.
3. Unlike other SNN SE networks, our models operate directly on raw waveform speech signals, eliminating any overhead from pre- and post-frequency domain processing.
4. Both of our SNN models are composed exclusively of spiking neurons with no involvement of conventional ANN neurons, ensuring a fully spiking architecture throughout the networks.

## 2 Background and related work

### 2.1 ANNs for speech enhancement

Numerous models have been developed to address the SE problem. Some operate in the frequency domain, requiring additional overhead of pre- and post-processing steps to transform audio frames between the time and frequency domains. Other models operate directly in the time domain, differing in complexity and architectural design. In this work, we focus on two time-domain architectures, Wave-U-Net and Conv-TasNet, which have demonstrated competitive performance.

Wave-U-Net (Stoller et al., 2018) is a fully convolutional architecture that employs an encoder-decoder design with equal depth in the encoder and decoder, connected by skip connections linking corresponding layers. The encoder functions as a multi-scale feature extraction pipeline, capturing speech information through a series of downsampling blocks that progressively reduce the input dimensions. The decoder is responsible for reconstructing the signal and generating an enhanced speech frame. Skip connections further enhance the decoding process by transferring information between the encoder and decoder. At each decoding level, the encoded feature map is concatenated with the output of the corresponding decoder level, helping to preserve finer details that may have been lost during the downsampling process.

Conv-TasNet (Luo and Mesgarani, 2019), a more complex architecture than Wave-U-Net, adopts an encoder-separator-decoder design, with the separator serving as the core of the model. The encoder consists of a convolutional layer that encodes the speech frame into a two-dimensional spectral-like feature map. This feature map is passed to the separator, which comprises a series of dilated depthwise separable convolutional blocks with progressively increasing dilation factors. These expanding dilations enable the model to capture long-term dependencies in the signal, while the depthwise separable convolutions enhance computational efficiency. In speech enhancement tasks, the separator's primary role is to estimate a noise mask within the signal. This estimated mask is applied to the encoded feature map from the encoder, which is then passed to the decoder to reconstruct the predicted enhanced signal.

### 2.2 SNNs for speech enhancement

The shift of research attention towards using SNNs for SE is very recent, resulting in limited studies in this area. Riahi and Plourde (2023) proposed a spiking U-Net encoder-decoder architecture for SE, which incorporates skip connections between corresponding encoder and decoder layers. This model operates in the frequency domain, where the input audio frame undergoes a Short-Time Fourier Transform (STFT) to produce its spectral representation, followed by a fully connected conventional ANN layer for signal masking.

Spiking-FullSubNet, developed by Hao et al. (2024), is also designed to operate in the spectral domain. This model incorporates Gated Spiking Neurons (GSNs), which dynamically adjust membrane potentials using a variable decay factor that adapts to variations in the noise levels of the audio signal. The architecture employs both full-band and sub-band processing. The full-band model processes the noisy input spectrogram, capturing global dependencies and partitioning frequencies. These partitioned frequencies are then processed by sub-band models, which capture intra-band dependencies within specific frequency bands. The outputs from the sub-band models are further filtered before generating an enhanced spectrogram, which is transformed back into an enhanced audio signal using the inverse STFT.

Sun and Bohte (2024) proposed DPSNN, a time-domain masking method with an encoder-separator-decoder architecture. The encoder transforms audio frames into 2-D feature maps, which are processed temporally by the separator. The separator uses spiking neurons in convolutional and recurrent layers to generate a spiking mask, later converted into a binary mask by a conventional ANN layer. This mask refines the encoded features to reconstruct enhanced audio frames. It should be noted that DPSNN uses non-spiking neurons in its encoder and decoder, which may pose limitations in hardware implementation and energy efficiency.

### 2.3 SNN training

Training SNNs can be approached in several ways. They can be trained from scratch through supervised learning. However, the non-differentiable nature of spikes makes conventional backpropagation inapplicable. To address this, surrogate gradient algorithms (Li et al., 2021; Neftci et al., 2019) were developed. These algorithms address the differentiability issue by approximating the activation function with a differentiable alternative during backpropagation.

SNNs can be trained through unsupervised learning based on Spike-Timing-Dependent Plasticity (STDP) (Masquelier and Thorpe, 2007; Diehl and Cook, 2015; Lu and Sengupta, 2024). This learning rule follows the principle: “neurons that spike together, wire together.” Synaptic weights of the neurons are adjusted based on spike timing, which allows temporal learning of the network from the input. However, STDP is computationally expensive and achieving a stable weight convergence remains a significant challenge.

SNNs can also be obtained through converting fully trained ANNs. This approach began with early methods by Pérez-Carrasco et al. (2013), who used ANN weights to optimize SNNs with leakage and refractory periods. Cao et al. (2015) advanced this by removing these periods and linking ReLU to spiking activation. Diehl et al. (2015) further improved the approach with weight normalization and reset-by-subtraction, achieving strong performance on MNIST.

Alternative methods introduced temporal coding, with Rueckauer et al. (2017) refining SNN modeling through enhanced weight normalization, biases, batch normalization, max pooling, and static rate input encoding. Sengupta et al. (2019) focused on iterative threshold updates for deeper residual networks, achieving reduced conversion loss but requiring longer simulations. Ho and Chang (2021) contributed by developing Trainable Clipping Layers (TCL) to directly train thresholds, improving activation control and overall performance.

## 3 Method

The speech enhancement problem can be perceived as removing unwanted background noise and other distortions from a recorded speech signal. The goal of SE is to produce a cleaner version of the speech signal that is either more comprehensible to human listeners or more suitable for further processing by automatic systems. Mathematically, the input signal can be expressed as follows:


$$\begin{array}{l} y\left(x\right)=s\left(x\right)+v\left(x\right) \end{array}$$

where *y*(*x*) is the observed noisy speech signal, *s*(*x*) is the clean speech signal, and *v*(*x*) represents the additive noise, which can originate from various sources such as environmental sounds (e.g., traffic, background conversations), imperfections in recording equipment, or acoustic reverberation. This noise degrades the clarity of the speech signal, posing significant challenges for systems reliant on clean audio inputs, such as speech recognition and speaker identification systems.

The primary objective of speech enhancement model *f*(·) is to estimate or reconstruct the clean speech signal *s*(*x*) from the noisy observation *y*(*x*). The enhanced signal ŝ(*x*), representing the estimated clean signal, can be expressed mathematically as:


ŝ(x)=f(y(x))

### 3.1 Proposed three-stage hybrid ANN-SNN fine-tuning for SE

**Figure 1:**
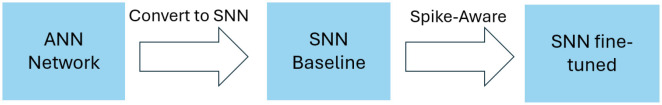
3-stage SNN fine-tuning pipeline.

In this work, we adopt a three-stage framework to speech enhancement, similar to the model we previously proposed for image segmentation (Baltes et al., 2023). As illustrated in Figure 1, the first stage of our framework involves training an SE ANN to convergence, ensuring it achieves peak performance. In the second stage, the trained ANN weights are used to initialize a baseline SNN. This baseline SNN is created as an equivalent spiking version of the ANN, ensuring that each layer in the ANN is accurately mapped to a corresponding layer in the SNN. Serving as a robust starting point, this baseline SNN undergoes fine-tuning of its weights to further enhance performance.

**Figure 2:**
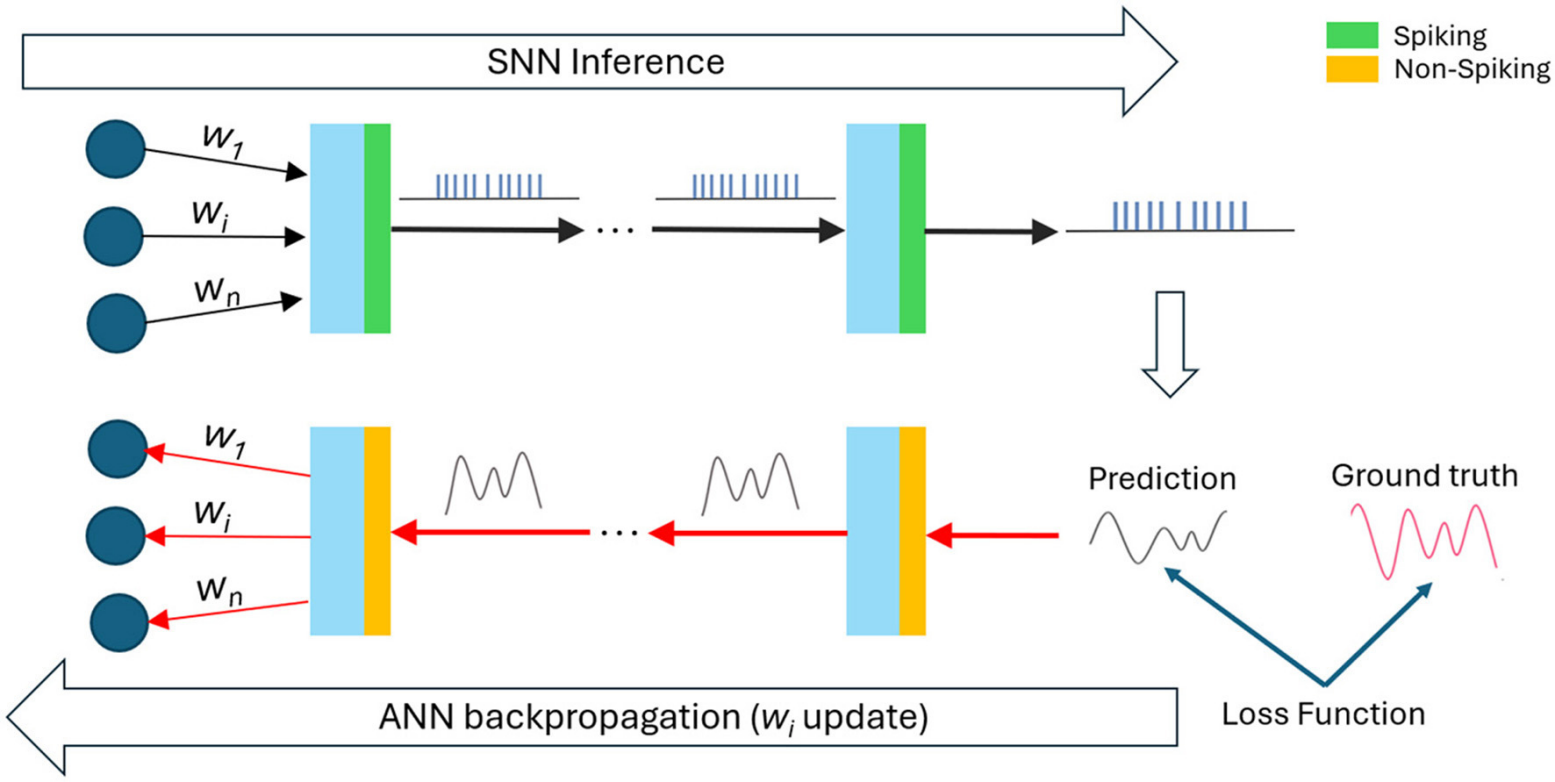
An illustration of the forward and backward passes during spike-aware training.

The fine-tuning process employs a *hybrid ANN-SNN training* strategy, also known as *Spiking aware training*, which involves alternating forward and backward passes as illustrated in Figure 2. The forward pass performs SNN inference and computes the loss between SNN outputs and the ground truth, while the backward pass uses conventional backpropagation to update the weights of the network. During the backward pass, the spiking activations are replaced with their non-spiking equivalents to facilitate gradient computation. The ANN backward pass ensures an efficient weight updates and a smooth gradient flow due to avoiding the non-differentiability issue that SNNs have due to undefined spike derivations.

#### 3.1.1 Spiking neuron model

*Hybrid ANN-SNN* follows a spiking quantization algorithm (Voelker et al., 2020) that translate arbitrary activation functions into their spiking equivalents as described by Algorithm 1. For a time-step *t*, let *x*_*t*_ be the input to an arbitrary activation function, where *x*_*t*_ is the neuron input multiplied by the synaptic weight, such that the spiking output rate is *y*_*t*_ = *f*(*x*_*t*_) in a specific time window ω with static state *v*_*t*_, where *t*>0. The output spiking rate *y*_*t*_ is determined by the Algorithm 1.

**Algorithm 1:**
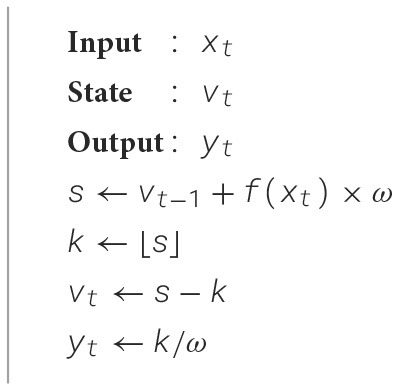
Spiking neuron quantization for an arbitrary activation function.

Following Algorithm 1, the neuron type is determined by the activation function *f*(*x*_*t*_). For example, Leaky-Integrated-and-Fire (LIF) neuron can be implemented by setting *f*(*x*_*t*_) to the time-averaged response curve. Furthermore, the algorithm is equivalent to the Integrate-and-fire neuron (IF) without a refractory period when *f*(*x*_*t*_) = *max*(*x*, 0) and i.e., ReLU and ω = 1, with membrane voltage *v*_*t*_ normalized to [0, 1). The ω parameter allows the neuron model to have multiple spikes per time-step, which can be interpreted as a quantization rate-based neuron.

In our work, we used IF neuron for all convolutional operations, and *f*(*x*_*t*_) = *x*_*t*_, i.e., linear activation, for the output layers in each model. Spiking neurons with linear activations provides a direct, proportional relationship between input and spiking activity, making it suitable for tasks where a linear mapping between input and output is needed.

### 3.2 Proposed SNN models for SE

In this work, we apply the proposed three-stage and hybrid ANN-SNN training framework to Wave-U-Net (Stoller et al., 2018) and Conv-TasNet (Luo and Mesgarani, 2019), two well-established architectures for speech enhancement tasks that operate directly in the time domain. We name our models SNN-Wave-U-Net and SNN-ConvTasNet, respectively.

#### 3.2.1 SNN-Wave-U-Net

**Figure 3:**
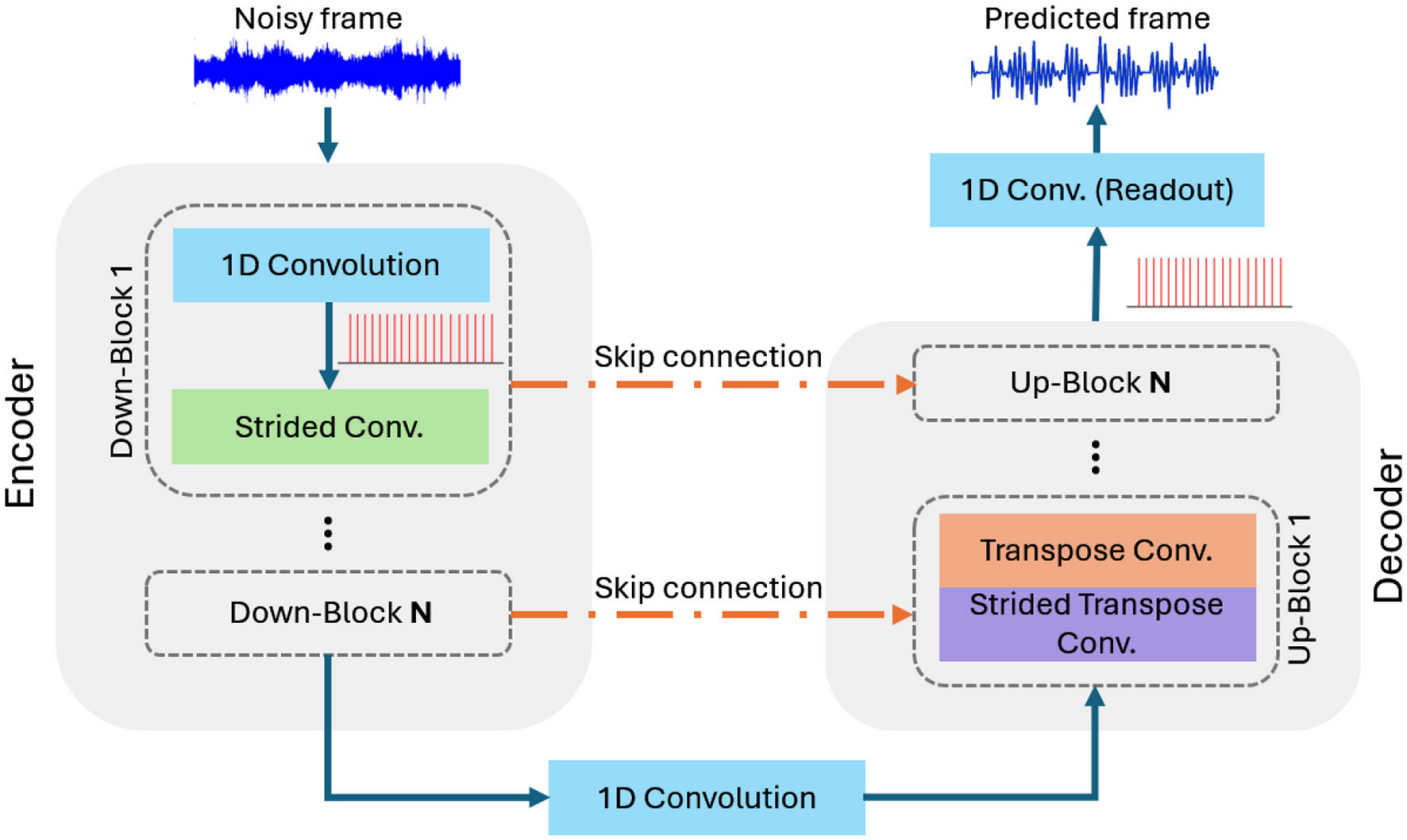
The encoder and decoder architecture in SNN-Wave-U-Net.

Our SNN-Wave-U-Net is a variation of the ANN-based Wave-U-Net. As illustrated in Figure 3, it adopts an encoder-decoder architecture that employs IF neurons for all layers within the encoder and decoder. Notably, the neurons do not have a refractory period, allowing them to spike multiple times within a predefined time window (ω). The spikes generated during (ω) are collected and processed by the subsequent layer. These neurons are referred to as quantization rate neurons. The output readout convolution layer consists of spiking neurons with a linear activation function. The network uses *direct input encoding* (Diehl and Cook, 2015) to convert the noisy input mixture into spikes within the first layer of the encoder. This layer functions both as a spike generator and as a feature extractor. Direct input encoding has been employed in various SNN applications, including image classification and speech enhancement (Diehl and Cook, 2015; Rathi and Roy, 2021; Riahi and Plourde, 2023).

The encoder of SNN-Wave-U-Net uses multi-scale downsampling blocks to extract speech features. Each block has a 1D-convolution layer for feature extraction and a strided convolution with a stride of 2 to halve the input size, improving efficiency and allowing deeper networks. The output of each block is a 2D feature map with encoded spike sequences. The final downsampling block's output is refined by a bottleneck 1D-convolution layer before decoding. The decoder uses upsampling blocks with 1D-transpose convolutions for signal reconstruction and strided transpose convolutions (stride of 2) to gradually double the feature map size, restoring the signal's original dimensions.

The skip connections between the encoder and decoder are implemented by concatenating the 2D feature maps from both at each level. These connections provide additional information that helps the decoder reconstruct the signal more accurately, resulting in a cleaner signal prediction. Finally, the decoder's output is processed by a 1D-convolution readout layer, where each point in the reconstructed audio signal is generated by counting the spikes emitted by the corresponding neuron in this layer.

To describe the model parameters, our SNN-Wave-U-Net has eight downsampling blocks in the encoder and eight upsampling blocks in the decoder. The encoder's 1D-convolutional layers use a kernel size of 15, while the strided convolutional layers have a kernel size of 2. The number of filters in these layers increases in multiples of 24 as the network goes deeper. Similarly, the decoder's upsampling blocks use strided transpose convolutions with a kernel size of 2 and a stride of 2, followed by transpose convolutions with a kernel size of 5. The number of filters in the upsampling layers also follows multiples of 24, increasing as the network moves up through the decoder. Finally the readout convolutional layer utilizes one filter of size 3.

We train our SNN-Wave-U-Net using the proposed three-stage hybrid SNN fine-tuning pipeline. As a preprocessing step, noisy audio tracks are divided into equal-length frames for network processing. First, an identical ANN Wave-U-Net with the same layer parameters is trained to convergence. The trained ANN weights are then transferred to the corresponding layers of the SNN-Wave-U-Net, creating a baseline SNN. Finally, the SNN-Wave-U-Net undergoes fine-tuning with spike-aware training to improve performance. Our SNN-Wave-U-Net utilizes one time step for input processing.

#### 3.2.2 SNN-ConvTasNet

**Figure 4:**
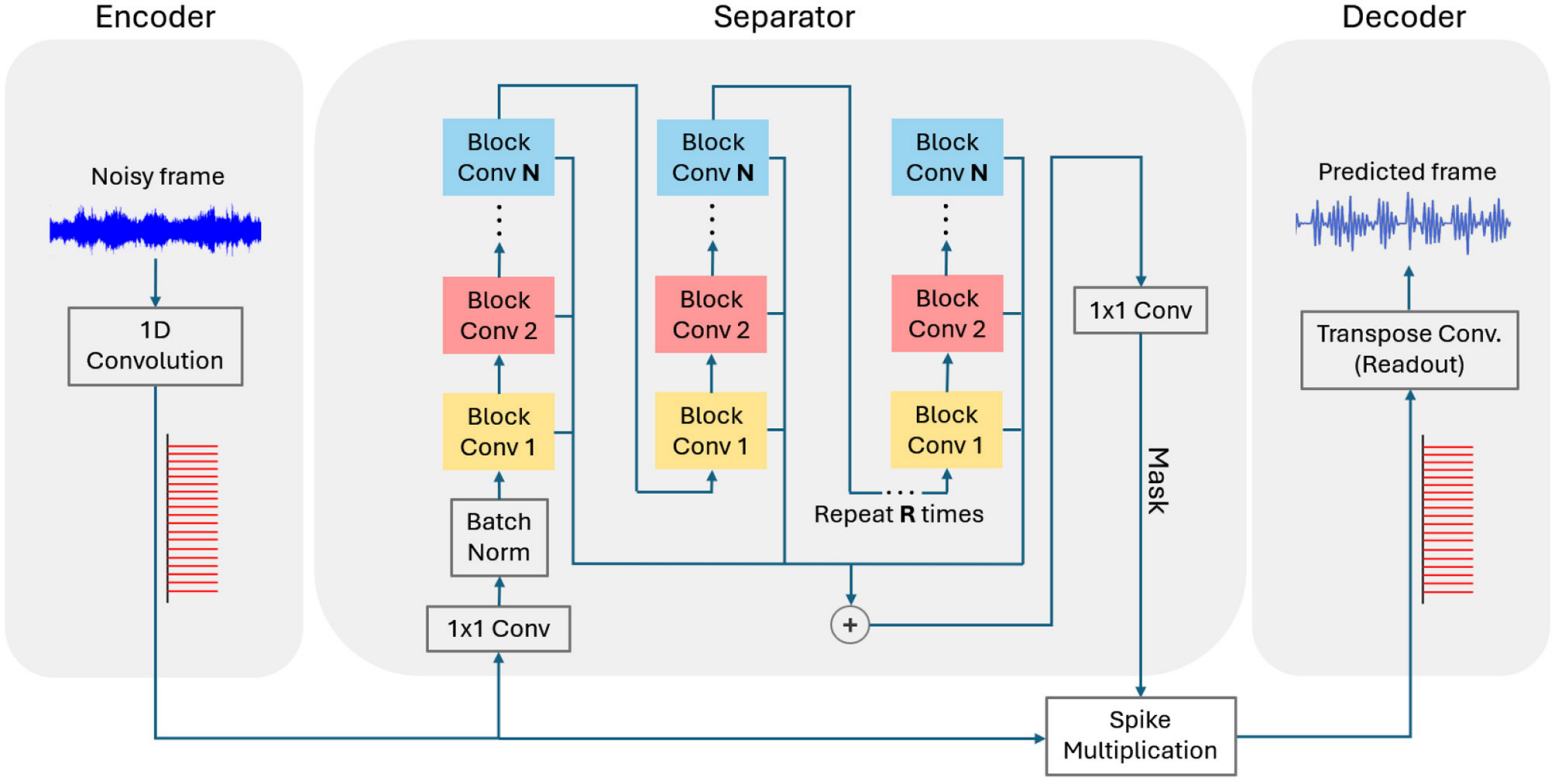
Encoder-separator- decoder of SNN-ConvTasNet architecture. Each color in the separator block represents a dilation factor within the group convolution layer increasing progressively from 2^1^ to 2^*N*^.

ConvTasNet is an encoder-separator-decoder architecture (Luo and Mesgarani, 2019). Our SNN-ConvTasNet is developed as a spiking-friendly adaptation of a modified version of the ANN ConvTasNet. These modifications were made to ensure compatibility between ANN and SNN components while maintaining comparable performance. For example, PReLU was replaced with ReLU, layer normalization with batch normalization, and the multiplication operation with an equivalent synaptic weight multiplication layer. The architecture of SNN-ConvTasNet, as shown in Figure 4, uses IF neurons for all layers except the readout layer, which employs spiking neurons with a linear activation function. None of the neurons have a refractory period.

Similar to SNN-Wave-U-Net, our SNN-ConvTasNet uses IF neurons and *direct input encoding* to convertthe noisy input mixture into spikes within the encoder layer. The encoder consists of a single strided convolution layer that produces a 2D feature map, with each point in the map represented as a sequence of spikes. This feature map effectively serves as the spectral equivalent of the input signal. The encoded feature map is then processed by the separator, which predicts a mask to be applied to the encoded feature map. The resulting masked feature map is subsequently used by the decoder to reconstruct the signal.

**Figure 5:**
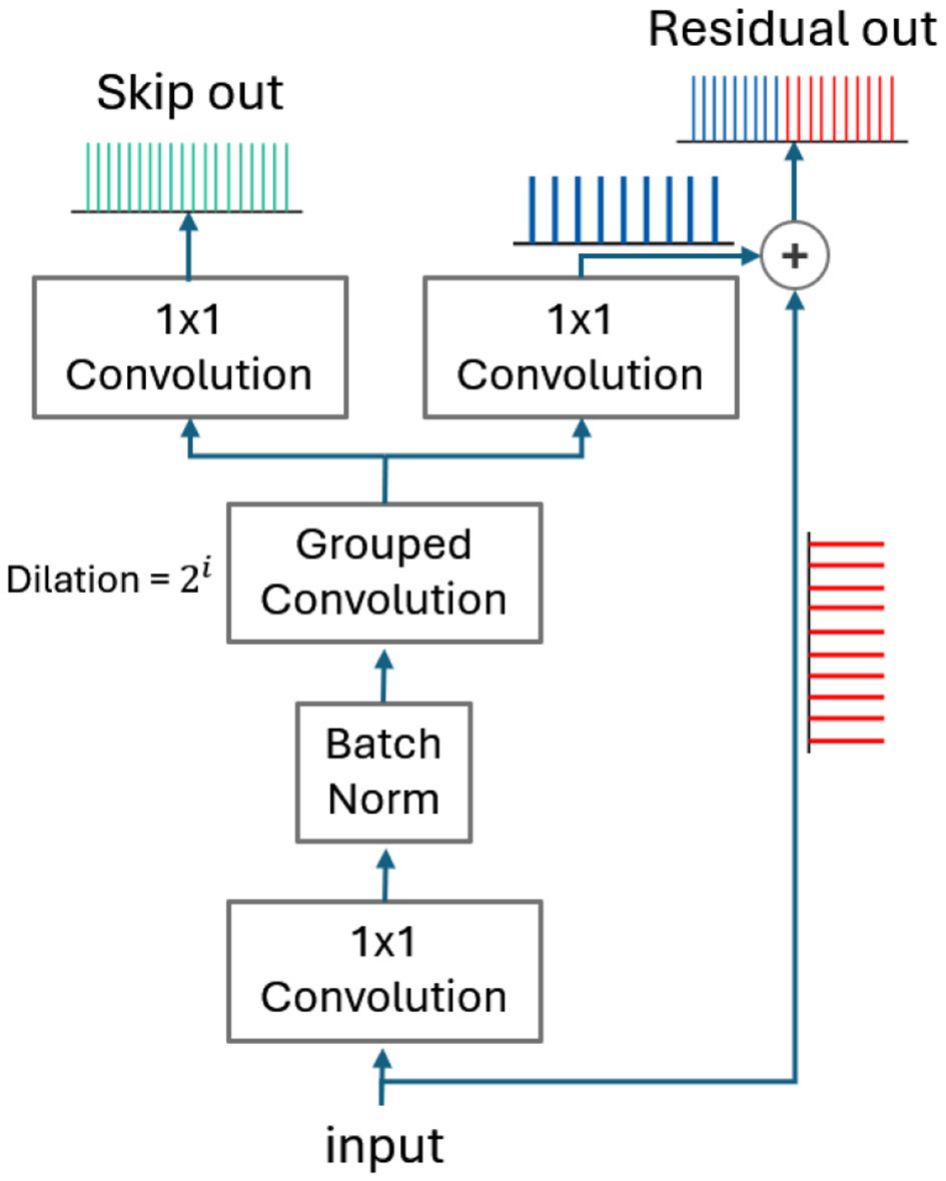
An illustration of a block convolution architecture inside the separator and how point-wise addition is performed on spike trains in the feature map.

The separator consists of *N* convolution blocks repeated *R* times. Each block uses dilation factors that progressively increase as powers of 2, expanding the receptive field to better capture complex audio patterns and improve mask prediction accuracy. As shown in Figure 5, each block includes a 1 × 1 convolution followed by a group convolution layer, which reduces computational complexity while maintaining efficient pattern learning. The group convolution layer produces two outputs: residual and skip feature maps, via separate 1 × 1 convolution layers. The residual output is created by adding the block's input to the 1 × 1 convolution output and is passed to the next block. The skip outputs from all blocks are summed together to form a 2D feature map. This combined map goes through a final 1 × 1 convolution to generate the mask for the separator.

The resulting mask is applied to the encoded 2D feature map through point-wise multiplication in the spike multiplication layer. The masked feature map is then passed to the decoder for signal reconstruction. The decoder consists of a single spiking 1D-transpose convolution layer with spiking neurons and linear activation. The neurons' spike counts are used to reconstruct the enhanced signal. The same three-stage hybrid SNN fine-tuning pipeline is utilized to train our SNN-ConvTasNet.

To summarize our SNN-ConvTasNet architecture parameters, the encoder uses a 1D convolution with a kernel size of 16, 256 filters, and stride factor of 8. The separator contains three blocks per repetition, with two repetitions, using dilated convolutions with dilation rates of 2^*j*^ and an expand ratio of 2. Each block has residual and skip connections with 256 channels. The dilated convolutions also utilize group convolutions, where the number of groups is equal to 512. The separator output is generated using a 1D-convolution with 256 filters and kernel size 1. As shown in Figure 4, the separator output is multiplied with the encoder output using a multiplication layer its detailed structure is presented in Section 3.2.3. Finally, the decoder reconstructs the waveform using a 1D-transposed convolution with a kernel size of 16, stride 8, and a single output filter.

#### 3.2.3 Spike multiplication and spike addition

**Figure 6:**
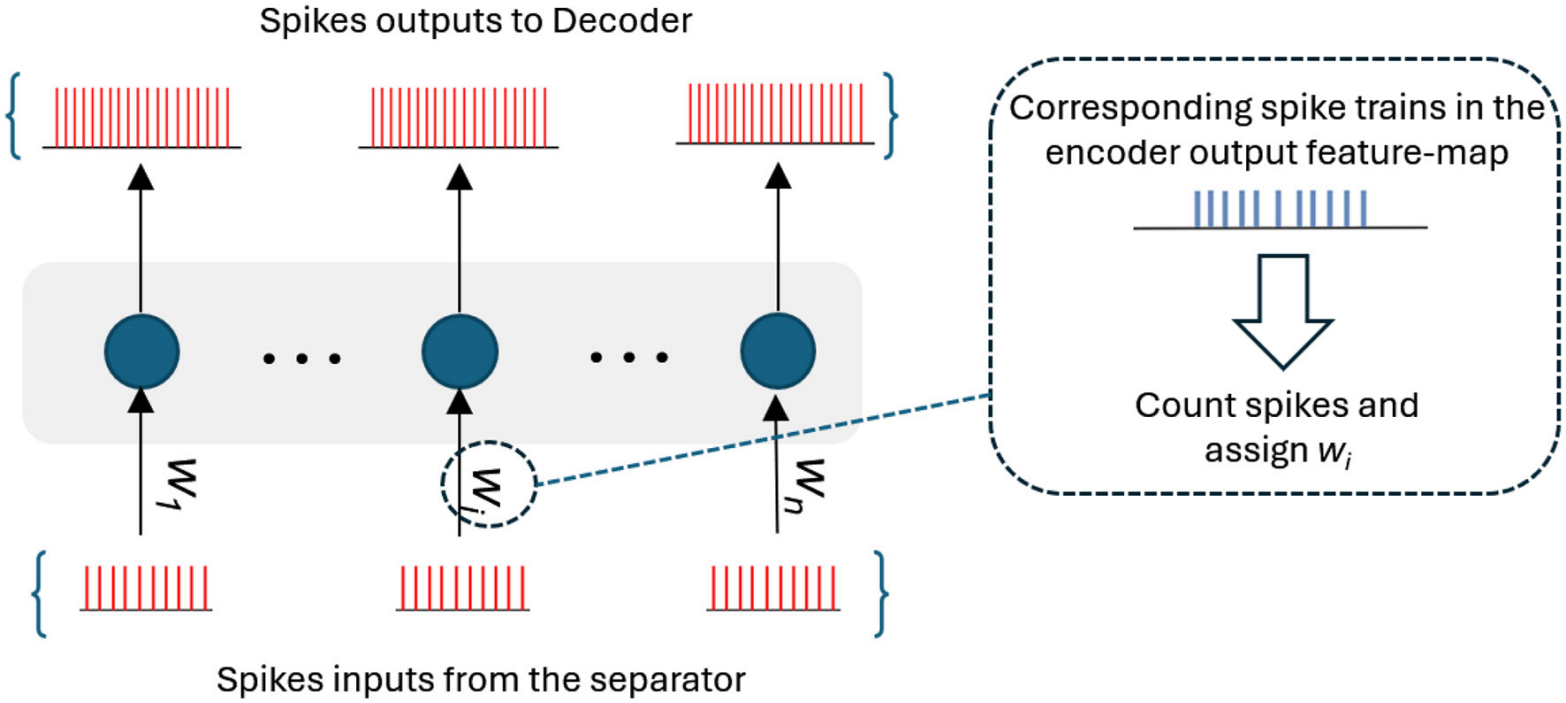
An illustration of a multiplication layer in SNN-ConvTasNet model.

In our SNN-ConvTasNet, point-wise spike multiplication between feature maps is a challenging task. To address this, we design a custom convolutional layer inspired by the concept of synaptic weight multiplication. This layer utilizes a single convolutional operation with one kernel, where the kernel size *n* is configured to match the flattened dimensions of the input 2D feature map. Based on Algorithm 1, the output spike count is determined by the floor of the membrane potential from the input layer, scaled by ω, the predefined time interval for spike collection. To align with this, the kernel weights *w*_*i*_ of the multiplication layer are initialized to the calculated number of spikes generated by the *i*th encoder neurons as shown in Figure 6. The output spikes from the separator are then used as input to the multiplication layer. This results in generating new spike trains from the multiplication layer that correlates with the point-wise multiplication of each point in the input feature map and its corresponding point in the encoder feature map which is represented as a kernel weight in the layer. Finally, the resulting spiking feature map is reshaped back to the original size of the input maps prior to flattening.

Moreover, we conclude from Algorithm 1 that the number of spikes generated by the IF neuron is proportional to the input *x*_*t*_, establishing it as a rate-based neuron quantizer. Consequently, the spike addition operation can be achieved by concatenating the respective spike trains together as shown in Figure 5. In our SNN-ConvTasNet, we perform a point-wise addition operation along 2D-feature maps, where each point in the feature map is represented by spike trains.

## 4 Experiments and results

In this section, we conduct experiments to evaluate the effectiveness of our proposed three-stage hybrid ANN-SNN fine-tuning framework, based on SNN-Wave-U-Net and SNN-ConvTasNet. We first describe the datasets, training strategy, and evaluation metrics. Then, the results of our model and the corresponding baseline models are compared and analyzed.

### 4.1 Data

Our experiments are conducted on two datasets: *Noisy VCTK* and *TIMIT with speech-shaped noises (SSNs)*. **Noisy VCTK** dataset (Valentini-Botinhao et al., 2016, 2017) comprises nearly 400 sentences spoken by 30 different speakers. For our experiments, we divided the dataset into 28 speakers for training and 2 speakers for testing and evaluation. The noise signals in the VCTK dataset are taken from the Demand dataset (Thiemann et al., 2013), which includes two artificially generated and eight naturally recorded noise types, each presented at four different SNR levels (0, 5, 10, and 15 dB). The original audio files have a sampling rate of 48 kHz. As a preprocessing step, we downsampled all audio tracks to 16 kHz for our task.

**TIMIT with SSNs** dataset is modified from the TIMIT dataset (Garofolo et al., 1993). The SSN noise is generated by taking broadband noise and filtering it to match the long-term frequency spectrum of the TIMIT sentences, the noise SNR levels are set to –6, –3, 0, +3, and +6 dB. TIMIT consists of 630 speakers with 8 different accents. We split this dataset as 20,790 utterences for training, 2,310 for validation, and 8,400 for testing. Similar to noisy VCTK data samples, all utterences have been downsampled to 16 KHz as a preprocessing step.

### 4.2 Training and evaluation

For SNN-Wave-U-Net, we first trained an ANN Wave-U-Net with the same layer architecture. The training was conducted using the ADAM optimizer (Kingma, 2014) with a learning rate of 0.001, an input size of 1 second, and the SNR loss function. The SNR loss function penalizes the difference between the enhanced signal and the ground truth clean signal, aiming to maximize the signal-to-noise ratio. The SNR loss function is formulated as follows:


$$\text{SNR Loss}=-10\cdot \log_{10}\left(\frac{||s_{\text{gt}}{||}^{2}}{||s_{\text{gt}}-s_{\text{enhanced}}{||}^{2}}\right)$$

where:

- *s*_gt_: Clean groundtruth signal.
- *s*_enhanced_: Enhanced signal produced by the model.
- ||·||^2^: Signal energy, typically calculated as the sum of squares.

After training the ANN model, we established a baseline SNN by transferring the weights from the ANN to the SNN-Wave-U-Net architecture. In the final step, SNN-Wave-U-Net is fine-tuned using hybrid ANN-SNN training with the SNR loss function, the Adam optimizer with learning rate of 0.0001, input frame size of 16,384 which represents approximately 1s of audio. For hyper-parameter ω, we performed a grid search over values [0.0001,1] and increase by a factor of 10 at each step to identify the optimal value. We set our IF neuron time step window of ω = 0.001. Each audio frame is processed in a single time step.

A similar approach was used to develop the SNN-ConvTasNet. First, an ANN-based ConvTasNet with an identical architecture was trained using the SNR loss function and the Adam optimizer with a learning rate of 10^−4^. The baseline SNN model was then initialized by transferring weights from the trained ANN. Finally, the SNN-ConvTasNet was fine-tuned using spike-aware training with the SNR loss function, the Adam optimizer with a learning rate of 10^−5^. We set the IF neuron time step window of ω = 1, selected via the same grid search criteria as of SNN-Wave-U-Net. Similar to SNN-Wave-U-Net, the input frame size is 16,384, and processed in one time step.

To evaluate our work, we used both perceptual metrics, such as STOI and PESQ, and objective metrics, including Signal-to-Noise Ratio (SNR), Segmented Signal-to-Noise Ration (SSNR), and Scale Invariant Signal-to-Noise Ratio (SI-SNR). In addition, we utilized the DNSMOS score, which provides three key metrics: speech signal quality (SIG), background noise quality (BAK), and overall audio quality (OVRL). In the context of speech enhancement, the SIG score reflects changes in speech quality due to processing, though most denoising algorithms typically result in minimal improvement in this score compared to the unprocessed signal. The BAK score indicates the level of noise present, where a significant improvement is expected after speech enhancement. Lastly, the OVRL score offers a general assessment of audio quality, not simply an average of SIG and BAK, but rather a holistic evaluation.

### 4.3 Results and analysis

The models evaluated include the original ANNs, the SNN models converted from the ANNs, and the fine-tuned SNN models. We performed individualized comparisons for Wave-U-Net and ConvTasNet, respectively.

Table 1 summarizes the experimental results of the models applied to the noisy VCTK test data. The *Noisy* column represents the statistics of the test data, the *Wave-U-Net* column presents the performance of the trained ANN model, the *Converted SNN* column shows the performance of converted (baseline) SNN with ANN weight transfer, and finally, the *SNN-Wave-U-Net* column shows the results for the fine-tuned SNN. All SNN predictions are obtained using one time-step processing.

**Table 1:** Evaluations of ANN Wave-U-Net, baseline SNN, and fine-tuned SNN on the VCTK test data.

**Metric**	**Noisy**	**Wave-U-net**	**Random**	**Converted SNN**	**SNN-Wave-U-net**
SNR	8.45	**15.41**	0.06	8.37	14.97
SSNR	1.68	**7.21**	–0.17	–0.30	6.72
SI-SNR	8.45	**15.64**	–14.24	9.03	15.33
STOI	**0.92**	0.91	0.71	0.83	0.91
PESQ	1.97	**2.04**	1.03	1.23	1.85
CSIGS	**3.34**	3.26	1.86	3.29	3.22
CBAKS	3.12	**3.97**	2.47	2.41	3.61
COVLS	2.69	**2.98**	1.44	2.30	2.79

Bold values indicate the best performance model on the corresponding evaluation metric.

The items in bold font represent the best performance among the competing models. A performance gap can be observed between the converted SNN and the ANN Wave-U-Net, indicating that the converted SNN, obtained through weight transfer, is not sufficient on its own. Additionally, we include results from a *random* SNN, where weights are assigned randomly. Compared to the random SNN, our weight transfer approach provides a significantly better starting point for fine-tuning, outperforming training from scratch. It is evident that our SNN-Wave-U-Net outperforms the converted baseline SNN, demonstrating the effectiveness of the fine-tuning step. Finally, we note that our SNN-Wave-U-Net achieves performance comparable to that of the ANN Wave-U-Net.

However, we see the models perform slightly lower in terms of STOI but still comparable. Furthermore, we see no improvement over CBAKS scores on this dataset. The reason can be conveyed to the struggle of CSIG metric with *dynamic noises* present in the Noisy VCTK dataset which fluctuates overtime. CSIG primarily focuses on signal fidelity, comparing the enhanced signal with a clean reference signal, penalizing any distortion to the signal that may occur during speech enhancement. Thus, when the background noise varies rapidly, as is the case in the Noisy VCTK dataset, even minor artifacts or aggressive noise suppression can lead to significant penalties in CSIG.

Table 2 summarizes the experimental results of the models on TIMIT with SSNs dataset. Similar to the noisy VCTK experiments, a performance gap is demonstrated between the baseline SNN and the ANN Wave-U-Net. Our fine-tuned SNN-Wave-U-Net, on the other hand, outperforms the ANN Wave-U-Net in most metrics except for PESQ. This observation may be explained by the robustness of spiking networks to gaussian noise present in our noisy TIMIT dataset (Li et al., 2020). All SNN predictions are obtained using one time-step processing.

**Table 2:** Wave-U-Net and SNN-Wave-U-Net performance on TIMIT test data.

**Metric**	**Noisy**	**Wave-U-net**	**Converted SNN**	**SNN-Wave-U-net**
STOI	0.66	0.70	0.63	**0.73**
PESQ	1.11	**1.30**	1.10	1.26
SI-SNR	0.0015	3.42	2.98	**4.55**
SNR	–2.94	4.07	3.56	**4.82**
Segmented-SNR	–5.62	–0.73	–2.40	**–0.27**
CSIG	1.79	1.84	1.52	**1.91**
CBAKS	1.43	2.47	1.80	**2.53**
COVLS	1.35	1.51	1.28	**1.52**

Bold values indicate the best performance model on the corresponding evaluation metric.

Tables 3, 4 present the performance comparison of SNN-ConvTasNet on the VCTK and TIMIT test datasets, respectively. A similar performance gaps are noticed between the baseline SNN and ANN ConvTasNet on VCTK and TIMIT data evaluations, which highlights the necessity of the fine-tuning step.

**Table 3:** ConvTasNet and SNN-ConvTasNet performance on VCTK test data.

**Metric**	**Noisy**	**ConvTasNet**	**Converted SNN**	**SNN-ConvTasNet**
SNR	8.45	15.97	–9.84	**16.47**
SSNR	1.68	7.96	–5.22	**8.19**
SI-SNR	8.45	16.00	2.08	**16.53**
STOI	**0.92**	0.91	0.77	0.91
PESQ	1.97	2.09	1.15	**2.19**
CSIGS	**3.34**	3.10	2.52	3.10
CBAKS	3.12	3.77	1.52	**3.84**
COVLS	2.69	2.77	1.47	**2.80**

Bold values indicate the best performance model on the corresponding evaluation metric.

**Table 4:** ConvTasNet and SNN-ConvTasNet performance on TIMIT test data.

**Metric**	**Noisy**	**ConvTasNet**	**Converted SNN**	**SNN-ConvTasNet**
STOI	0.66	**0.751**	0.52	0.747
PESQ	1.11	**1.42**	1.05	1.21
SI-SNR	0.0015	5.87	–18.96	**6.13**
SNR	–2.94	5.09	–9.68	**5.40**
Segmented-SNR	–5.62	0.52	–7.1796	**1.08**
CSIG	1.79	2.03	1.05	**2.05**
CBAKS	1.43	3.06	1.13	**3.53**
COVLS	1.35	1.77	1.04	**1.83**

Bold values indicate the best performance model on the corresponding evaluation metric.

Table 3 shows that our fine-tuned SNN-ConvTasNet performs considerably well and achieves a superior performance compared to ANN ConvTasNet over SNR metric variants. However, both ANN and SNN-Convtasnet did not have an improvement over the STOI and CSIGS metrics. On the other hand, Table 4 shows the evaluation performance on TIMIT test data, our SNN-ConvTasNet performs comparably well compared to ANN ConvTasNet and it outperforms the ANN on SNR metric variants, and CBAKS score. The SNN-ConvTasNet achieves a noticeable denoising improvement on all evaluation metrics compared to the noisy signal statistics. All SNN predictions are obtained using one time-step processing.

Furthermore, SNN-ConvTasNet outperforms both ANN and SNN-Wave-U-Net across most of the evaluation metrics. This improvement can be attributed to the more complex separator module in SNN-ConvTasNet, which offers enhanced noise separation capabilities compared to Wave-U-Net.

In this study, we conducted models energy analysis to evaluate and compare the efficiency of our proposed SNN models with their corresponding ANN counterparts. The energy consumption results were obtained using the KerasSpiking energy package. For SNN models, KerasSpiking estimates energy consumption by simulating SNN spike activity and assigns a cost per spike. It monitors spikes at each layer to calculate energy usage and estimates the overall energy of the model in Joules. On the other hand, ANN energy is estimated by calculating the number of multiply-accumulate operations *MACs* for each layer based on its input, output, and kernel size. The total MACs are then used to estimate the energy consumption. The comparison of the average total energy consumption is summarized in Table 5. The number of parameters for the baseline models (Wave-U-Net and ConvTasNet) are also shown as a reference.

**Table 5:** Energy consumption comparison of ANN Wave-U-Net Conv-Tasnet and our corresponding fine-tuned SNN-Wave-U-Net and SNN-Conv-Tasnet.

**Model**	**Param no**.	**Total energy [Joules/inf]**
Wave-U-Net	2.60M	14.6
SNN-Wave-U-Net		4.63
ConvTasNet	2.65M	44.4
SNN-ConvTasNet		6.37

The results show that SNN-Wave-U-Net consumes 4.63 Joules per inference, representing a 3.2 × reduction compared to Wave-U-Net, which consumes 14.6 Joules per inference. Similarly, SNN-ConvTasNet achieves a significant energy reduction, consuming only 6.37 Joules per inference compared to 44.4 Joules per inference for ConvTasNet, resulting in a nearly 7 × reduction. These findings underscore the future of SNNs for energy-efficient processing and potential application on neuromorphic hardware.

## 5 Discussions and conclusions

In this work, we designed and fine-tuned SNN-Wave-U-Net and SNN-ConvTasNet, two spiking time-domain speech enhancement models composed entirely of spiking neurons, with no reliance on traditional ANN neurons. This design makes them well-suited for potential implementation on SNN hardware. Both spiking models demonstrated comparable or superior performance to their ANN counterparts, particularly on speech-shaped noise data, highlighting the promise of SNNs for speech enhancement applications.

We explored IF spiking neurons in this study, which are highly computationally efficient and simple to implement. While this model captures the core dynamics of spiking behaviors, it omits key aspects of biological plausibility, such as the refractory period and more complex synaptic dynamics (Lapicque, 1907). To improve biological realism, we plan to explore leaky integrate-and-fire (LIF) neurons, where the leakage term more accurately represents the gradual dissipation of charge across a neuron's membrane.

In future research, we aim to explore the latency of both models in greater detail and focus on optimizing their latency performance. Moreover, we plan to investigate variations of Wave-U-Net and ConvTasNet, paying close attention to design details that significantly impact latency. Furthermore, we will examine the implementations of different spiking neuron types to experiment the performance and efficiency of the models.

Furthermore, since the ultimate applications of speech enhancement include headsets, hearing aids, and video conferencing, we will investigate hardware implementation on portable devices. To this end, we aim to evaluate commercially available edge platforms, including SynSense (Yao et al., 2024), BrainChip (Posey, 2022), and Innatera (Ward-Foxton, 2021), as well as Intel Loihi 2 (Davies et al., 2021), which is accessible for academic use.

## Data Availability

The original contributions presented in the study are included in the article/supplementary material, further inquiries can be directed to the corresponding author.
